# Effectiveness of Tranexamic Acid in Reducing Hidden Blood Loss During Laparoscopic Sleeve Gastrectomy: A Randomized Clinical Trial

**DOI:** 10.3390/jcm14093010

**Published:** 2025-04-26

**Authors:** Ksawery Bieniaszewski, Monika Proczko-Stepaniak, Maciej Wilczyński, Piotr Nowicki, Justyna Bigda, Michał Szymański

**Affiliations:** Department of Surgical Oncology, Transplant Surgery and General Surgery, Faculty of Medicine, Medical University of Gdansk, 80-214 Gdansk, Poland; mproczko@gumed.edu.pl (M.P.-S.); mwilczynski@gumed.edu.pl (M.W.); pnowicki@gumed.edu.pl (P.N.); jbigda@gumed.edu.pl (J.B.); szymanski@gumed.edu.pl (M.S.)

**Keywords:** bariatric surgery, tranexamic acid, bleeding

## Abstract

**Background**: Tranexamic acid (TXA), an antifibrinolytic agent, has demonstrated efficacy in reducing bleeding across various surgical procedures. However, its role in bariatric surgery remains underexplored. This study aimed to evaluate the effectiveness of TXA in mitigating hidden blood loss following laparoscopic sleeve gastrectomy (SG). **Methods**: A single-center, single-blind, randomized, controlled trial was conducted at the University Clinical Center, Medical University of Gdańsk, Poland, between July 2022 and June 2023. A total of 238 patients undergoing SG were randomized to receive either TXA or no pharmacological intervention. The primary outcome was hemoglobin concentration in abdominal drainage post-surgery. Secondary outcomes included total blood loss, drainage volume, the need for blood transfusion, and postoperative complications. Statistical analyses were conducted using intention-to-treat and per-protocol strategies. **Results**: A statistically significant reduction in hemoglobin concentration in abdominal drainage samples was observed in the TXA group (*p* = 0.011). No significant differences were found in total blood loss, drainage volume, necessity for blood transfusions, or extended hospital stay between groups. **Conclusions**: While TXA administration may reduce the hidden blood loss effect, its general clinical significance appears questionable. Nonetheless, intraoperative TXA may be beneficial for a selected patient group with multiple preoperative disorders and risk factors. Further research is necessary to comprehensively assess the risks and benefits of TXA administration in bariatric surgery.

## 1. Introduction

Obesity and its metabolic complications are an increasing challenge for healthcare systems. Bariatric surgery is a well-established and safe method of treatment [1,2]. However, it is associated with adverse events (AEs) including nutritional deficiencies, anastomotic fistulas, gastrointestinal leaks, and hemorrhage [3,4,5]. Bleeding is one of the most common AEs—reported rates vary from 0.5% to 4% [6]. It is also associated with a significant increase in other AEs [7].

The implementation of an enhanced recovery after bariatric surgery (ERABS) protocol [8] resulted in the early mobilization of patients and a reduced length of hospital stay (LOS), therefore reducing the overall time of observation. Ensuring patient safety remains a critical consideration. Postoperative bleeding remains one of the most common AEs following sleeve gastrectomy (SG) [9] and is associated with higher morbidity and mortality [10]. The rate of early (30-day) rehospitalization is estimated at around 6% [11]. Bleeding is a common reason for readmission and may require urgent endoscopic or surgical management, undermining the cost-effectiveness of bariatric procedures [12,13,14]. Consequently, achieving optimal hemostasis is essential to decrease readmission rates and increase patients’ safety.

Hidden blood loss (HBL) phenomenon refers to a decrease in hemoglobin concentration during the perioperative period that is not associated with clinically visible bleeding. Even when overt hemorrhage is minimal during laparoscopic surgery, a measurable postoperative hemoglobin drop can occur due to internal or “hidden” bleeding into tissues or the peritoneal cavity. This concept was first proposed in 2000 by Sehat [15]. Although usually having no effect on the postoperative period, it can lead to unnecessary diagnostics and procedures, therefore prolonging the hospitalization.

The use of tranexamic acid (TXA) to decrease postoperative bleeding in bariatric surgery has been increasingly researched in recent years [16]. The use of TXA has been proven effective in reducing postoperative bleeding across various surgical procedures. TXA reduced the need for transfusions after surgery and for surgical interventions [17,18]. Owing to its mechanism of action, TXA may increase the risk of venous thromboembolism (VTE) [19,20,21,22]. Obesity itself is associated with a higher VTE risk, and undergoing bariatric procedure along with restricted oral fluid intake can additionally increase this risk [23].

Based on multi-center randomized trials, the use of TXA as an additional hemostatic factor during surgical procedures has proven to be effective [17]. However, no randomized trial has been conducted to evaluate its use in bariatric surgery.

The aim of this study was to evaluate the effectiveness of TXA administration in reducing the volume and hemoglobin concentration in abdominal drainage following SG.

## 2. Materials and Methods

### 2.1. Trial Design

This preliminary study was a single-center, single-blind, randomized, controlled, clinical trial with a parallel-group design and a 1:1 randomization ratio. The trial protocol is detailed in Supplementary Material S1.

### 2.2. Participants

Eligible participants were adults qualified for a bariatric procedure at the University Clinical Center, Medical University of Gdańsk, Poland. Qualification adhered to the International Federation for the Surgery of Obesity (IFSO) guidelines and the recommendations of the Bariatric Chapter of the Association of Polish Surgeons [24,25]. Recruitment occurred between 4 July 2022 and 28 June 2023.

The inclusion criteria were as follows:

- Age over 18;
- Qualification for SG;
- Obtained, informed consent for participating in the study.

The primary (preoperative) exclusion criteria were as follows:

- Use of anticoagulative agents in the perioperative period, including the following:
  ○ Indirect thrombin inhibitors (Fondaparinux, UFH, and LMWH in therapeutic doses);
  ○ Direct inhibitors of factor Xa (NOAC);
  ○ Direct thrombin inhibitors (Dabigatran);
  ○ Vitamin K antagonists (VKA: acenocoumarol, warfarin);
  ○ Platelet aggregation inhibitors (excluding ASA in doses of 75 mg per day);
  ○ P2Y12 receptor inhibitors.
- Known blood coagulation disorders (congenital or acquired);
- History of TXA allergy;
- Chronic kidney disease in stage ≥ G3;
- Chronic hemodialysis or history of hematuria;
- Seizures in medical history.

A written, informed consent was obtained from all the participants before the trial began. The trial was registered at www.clinicaltrials.gov (NCT06038981). The registration was performed during the trial on 8 September 2023. The Consolidated Standards of Reporting Trials (CONSORT) guidelines were followed [26]. The study design and protocol were approved by the Institutional Ethics Committee of the Medical University of Gdańsk (NKBBN/439/2022, approval date 14 November 2022). All procedures performed in this study involving human participants were in accordance with the 1964 Helsinki Declaration and its later amendments.

### 2.3. Interventions

Every patient received a preoperative dose of LMWH (40 mg of enoxaparin sodium) as a prophylaxis, according to local guidelines, 12 h before surgery. All qualified patients received a single dose of 1 g i.v. TXA bolus at a loading dose of 1 g over 10 min, within 10 min of the induction of anesthesia, preceding the skin incision. Such dosing allows for the maximum concentration of medication to be achieved throughout the procedure. All procedures were performed by qualified and certified bariatric surgeons following a highly standardized protocol. Preceding the end of the procedure, an abdominal drain was placed alongside the staple line to monitor blood loss in the postoperative period. Drainage placement was performed only for study purposes. On the first postoperative day, the volume of drainage was monitored, and a sample was taken to perform a complete blood count (CBC) in a single, local laboratory with an automated method of evaluation—the result of the test was noted. To standardize the conditions for monitoring blood loss, all peripheral blood samples were taken on the day of admission and at 5 a.m. on the first day after surgery. Patients were discharged from the hospital if the discharge criteria were met (detailed description in the standard hospitalization protocol, Supplementary Material S2). Within 30 days of randomization, the patients were assessed by a qualified physician during a follow-up appointment: a clinical examination for arterial and venous thromboembolism was performed, basic laboratory tests were ordered, and the Clavien–Dindo grade was evaluated [27].

### 2.4. Outcomes

The primary outcome parameter was the intraperitoneal postoperative blood loss, defined as the mean hemoglobin concentration in the drainage sample, measured through performing a CBC. The secondary outcomes were blood loss (defined as the difference between the preoperative and postoperative hemoglobin concentrations in the peripheral blood sample), the drainage volume in mL, the hemoglobin mass in drainage (evaluated by multiplying the drainage volume and hemoglobin concentration), the procedure time (minutes), the extended postoperative hospital stay (days), major surgical complications due to bleeding (defined as a Clavien–Dindo grade ≥ 3), and necessity for blood transfusion.

### 2.5. Sample Size

No data were available before this trial on the concentration of hemoglobin in postoperative abdominal drainage after an intravenous administration of TXA. Therefore, a pilot study was commenced. During the period from 4 July to 19 December 2022, 142 patients were randomized, from which 123 patients were enrolled in the analysis. The univariate analysis revealed an exponential distribution of the obtained data. The SAS^®^ power procedure was performed regarding such data distribution. It was shown that, for a sample size of approximately 100 subjects per group, a significant (*p* < 0.05) difference in hemoglobin concentration is expected, with the power of the test equal to 0.8. During a 12-month period, 238 patients were enrolled in the study. The interim analysis of data confirmed the intended power and significance for the difference in hemoglobin concentration between groups. Therefore, the trial was closed.

### 2.6. Randomization

This was a single-blind, randomized, controlled, clinical trial. After signing their informed consent, patients were not informed of their allocation to the control and test groups. A block randomization was used to allocate patients to different interventions based on the operating schedule of the center. Block size varied depending on the operating room availability. Patients were assigned to the control and test groups alternately on a weekly basis. To further reduce possible bias, patients were deemed qualified for operation by physicians who had no interaction with the physicians scheduling the operations. Moreover, a different physician was responsible for the randomization scheme and the data collection. Data analysis was performed by another independent physician from outside the center who was blinded to the allocation.

### 2.7. Statistical Methods

The collected data were analyzed according to both the intention-to-treat and per-protocol principles. Categorical data were presented as numbers and percentages of the analyzed group. Continuous variables were reported using mean and a 95% confidence interval for normally distributed data and as median with a 5th–95th percentile range otherwise. Statistical tests were performed using SAS Studio ver. 3.1.0. Group comparison for categorical variables was assessed using the chi-square test. ANOVA or Mann–Whitney U tests were used for normally or non-normally distributed continuous variables, respectively. The level of statistical significance was set at *p* < 0.05, 2-sided.

## 3. Results

**Figure 1:**
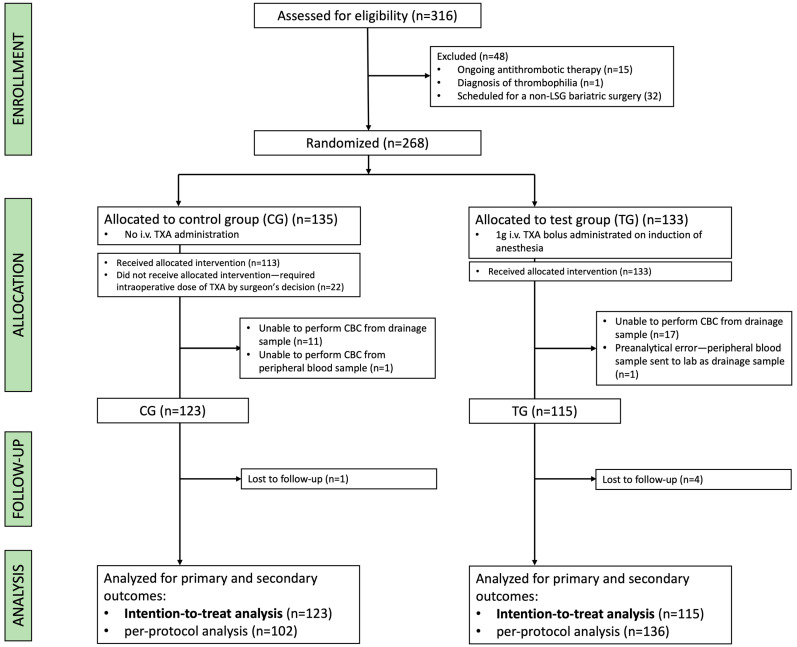
Study flowchart. TXA—tranexamic acid; CBC—complete blood count; LSG—laparoscopic sleeve gastrectomy.

Between July 2022 and June 2023, 316 patients were assessed for eligibility. In total, 268 eligible patients were randomly assigned to receive no pharmacological intervention (control group, CG) or TXA (test group, TG) (Figure 1).

Additionally, owing to missing crucial lab test results, 30 patients were excluded from the trial (CG = 12; TG = 18). Therefore, a total number of 238 patients (CG = 123; TG = 115) were included in the analysis. The obligatory 1-month follow-up visit was completed for 97.9% (*n* = 233) of the analyzed patients. Between 4 July 2022 and 28 June 2023, all the qualified patients underwent surgery. A total of 22 patients originally assigned to the control group (CG) required an intraoperative dose of TXA. All patients originally assigned to the test group (TG) received the allocated intervention. After 12 months of patient recruitment, the trial was concluded. The total data-gathering period ended on 4 August 2023. The baseline characteristics were comparable between both study groups (Table 1).

### 3.1. Intention-to-Treat Analysis

#### 3.1.1. Primary Endpoint

A statistically significant difference in the mean hemoglobin concentration in the drainage samples was found between the study groups in both the intention-to-treat (CG = 1.4 g/dL, TG = 0.7 g/dL, *p* = 0.011) and per-protocol analysis (CG = 1.7 g/dL, TG = 0.7 g/dL; *p* = 0.001) (Table 2).

#### 3.1.2. Secondary Endpoints

Table 2 presents an analysis of the patient data. The hemoglobin mass evaluated from the drainage sample differed between the study groups (CG = 0.4 g, TG = 0.2 g; *p* = 0.005). There was no difference in mean drainage volume (CG = 40 mL, TG = 40 mL; *p* = 0.187). Moreover, postoperative blood loss did not differ significantly between the groups (CG = 0.7 g/dL, TG = 0.5 g/dL; *p* = 0.103). No significant difference was found in the mean procedure time (CG = 55 min, TG = 54 min; *p* = 0.880). An extended postoperative stay, defined as a deviation from discharge on the first day after the procedure, was noted in seven patients in the control group; three patients in the test group required a prolongation of the hospital stay. There was no significant difference between the study groups regarding blood transfusions. In the control group, two patients required a transfusion of a total of five red blood cell packs and two freshly frozen plasma packs; in the study group, one patient required a transfusion of three red blood cell packs. No VTE or peripheral arterial thrombosis events were noted at the 1-month follow-up visit in both groups.

### 3.2. Subgroup Analyses

**Figure 2:**
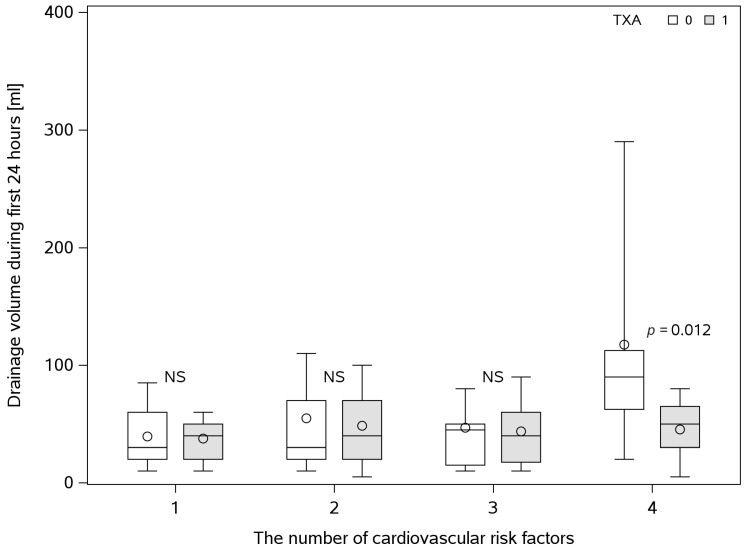
Box and whisker plot comparing abdominal drainage volume with and without TXA administration between subgroups, with an increasing count of metabolic syndrome components, where 1 stands for obesity, solely. The box and whisker plot shows quartiles of data where median and mean values are represented by the central box line and circle, respectively. The between-group comparison was performed using the Mann–Whitney U test. TXA—tranexamic acid.

As mentioned in the study protocol, because of clinical relevance, two additional subgroup analyses were performed. The first analysis, during the examination of patients on admittance to the hospital, considered the preoperative presence of metabolic syndrome components (obesity, arterial blood hypertension, type 2 diabetes, dyslipidemia, etc.) known to increase the general complication rate in bariatric surgery. The analysis was carried out in subgroups characterized by the sum of metabolic syndrome components. Patients with a higher number of obesity complications had a significantly smaller drainage volume after intraoperative TXA administration (Figure 2); however, no significant differences were noted between those subgroups in hemoglobin concentration and mass in drainage samples.

**Figure 3:**
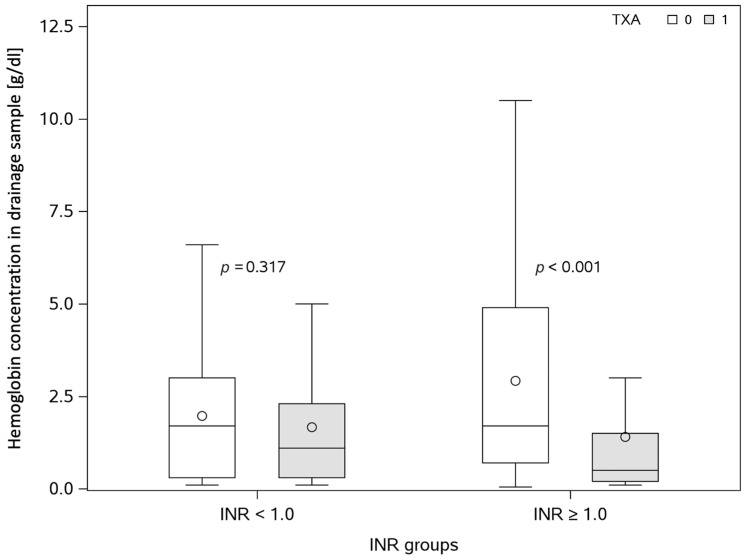
Box and whisker plot comparing hemoglobin concentration in abdominal drainage with and without TXA administration between subgroups with different INR value ranges. The box and whisker plot shows quartiles of data where median and mean values are represented by the central box line and circle, respectively. The between-group comparison was performed using the Mann–Whitney U test. TXA—tranexamic acid. INR—international normalized ratio of prothrombin time.

An additional subgroup analysis was performed considering preoperative INR values. Patients with INR < 1 did not have any significant differences in volume, hemoglobin concentration, and mass in the drainage sample. However, patients with the INR value > 1 had a significantly smaller hemoglobin concentration and mass in the drainage sample (Figure 3).

### 3.3. Adverse Events

The Clavien–Dindo classification was used during the obligatory 1-month follow-up visit to evaluate complications. Events considered as major complications (CD grade ≥ 3) were noted in six patients in the CG and three patients in the TG. All complications are listed in Supplementary Table S1. Additionally, there was no need for upper endoscopy for bleeding in either study group.

### 3.4. Per-Protocol Analysis

Both primary and secondary outcomes were analyzed as stated above for the patients allocated to the CG and TG according to the per-protocol principle. Results are presented in Table 2.

## 4. Discussion

The results of our study can be summarized as follows: TXA significantly reduced hemoglobin concentration in abdominal drainage samples after SG, indicating a reduction in hidden blood loss. We found no clinically meaningful differences in total blood loss, drainage volume, necessity for blood transfusions, or length of hospital stay between the two groups. Moreover, while TXA demonstrated potential benefits in reducing hidden blood loss, its clinical significance in routine practice remains questionable, and its use may be most beneficial in selected patient groups with multiple preoperative risk factors.

Our study aimed to assess the role of TXA in reducing hidden blood loss after SG, and the findings suggest that TXA has a measurable impact on postoperative blood loss, as reflected by hemoglobin concentrations and hematocrit in the drainage samples. In enhanced recovery protocols (ERABS), where patients are rapidly discharged, unrecognized intra-abdominal bleeding becomes a key concern. By monitoring HBL (via the abdominal drain hemoglobin content, in our trial), we aimed to detect even subtle differences in internal bleeding, thereby providing clinicians with an early insight into the potential bleeding source and reducing the risk of unnecessary imaging or interventions. This approach aligns with ERABS goals; minimizing hidden hemorrhage enhances patient safety and helps conserve healthcare resources by avoiding needless tests or prolonged observation.

The reduction in hemoglobin concentration and hematocrit was statistically significant in both the intention-to-treat and per-protocol analyses. This result supports the hypothesis that TXA may help mitigate HBL, a phenomenon that is not always evident clinically but may lead to unnecessary diagnostic procedures or prolonged recovery. In the literature, a reduction in postoperative hemoglobin level without surgical bleeding is frequently attributed to HBL [29,30]. This effect, which significantly increases cumulative blood loss, has been extensively documented in both orthopedic and abdominal surgical contexts [31,32,33]. The clinical implications of HBL may include an increased need for blood transfusions, extensive diagnostic evaluations, and prolonged LOS [34,35]. The ERABS protocol discourages the routine placement of postoperative abdominal drains, which may exacerbate the risk of hidden blood loss, and highlights the necessity for improved hemostatic strategies to support postoperative recovery [36]. Despite the observed reduction in drainage hemoglobin concentration, our findings do not align with previous studies that have demonstrated a reduction in blood loss following TXA administration [37].

The total blood loss did not differ significantly between the TXA and control groups (*p* = 0.103). Similarly, no significant differences were observed in the mean drainage volume, with both groups showing similar amounts of fluid in the drainage system post-surgery. These findings suggest that while TXA can reduce HBL, it does not appear to have a substantial effect on overall blood loss or the volume of drainage [38].

Our findings confirm that TXA significantly lowered the hemoglobin concentration and hematocrit in the drainage fluid—indicating reduced intraperitoneal bleeding—without changing the overall drainage volume. Simultaneous reductions in both hemoglobin concentration and hematocrit in drainage fluid suggest that TXA significantly altered the nature of the fluid. In practical terms, TXA seems to improve the nature of the fluid without influencing its quantity, suggesting less blood within the same amount of postoperative fluid. Other studies, including meta-analyses, have reported a positive effect of TXA on blood loss [39,40]; the lack of statistical significance in our study may be attributed to the difference in study design and limited study population.

Nevertheless, selecting HBL as the primary outcome allowed us to objectively quantify TXA’s hemostatic benefit. It provides a nuanced understanding of how TXA works—by curbing immediate microscopic bleeding—even if major clinical outcomes (e.g., overt hemorrhage or length of stay) remain unchanged in a setting with overall low bleeding risk. Such an endpoint is valuable for ongoing research because it captures the pharmacological effect of TXA in a quantifiable way.

Furthermore, this study found no significant differences in the incidence of major surgical complications, including the need for blood transfusions or prolonged hospital stay. Only a small proportion of patients in both groups required blood transfusions, with no major complications related to bleeding observed in either group. The observed reduction in hidden blood loss did not translate into differences in total hemoglobin drop or transfusion requirements in our cohort, implying that the clinical impact for these generally low-risk patients was limited [41,42,43].

The clinical implications of these findings are worth considering. While TXA can reduce HBL, its effect does not seem large enough to warrant its routine use for all bariatric surgery patients. Given the lack of substantial differences in total blood loss and clinical outcomes, the use of TXA should be reserved for specific patient populations who may benefit from it the most. In higher-risk subgroups (for instance, patients with pre-existing coagulation disorders, significant anemia, multiple obesity-related comorbidities, etc.), any reduction in hidden bleeding could be clinically pivotal, potentially preventing threshold drops in hemoglobin that might otherwise necessitate transfusion. Thus, although the absolute HBL in our study was small and clinically benign for most patients, focusing on this endpoint was justified as it illuminates TXA’s efficacy in enhancing hemostasis and supports its future use as a safeguard in ERABS protocols.

Our subgroup analysis indicates that patients with a higher number of metabolic syndrome components had a significantly smaller drainage volume, suggesting that TXA may be particularly beneficial in this higher-risk cohort. This subgroup may benefit the most from intraoperative TXA administration, as it reduces both the nature and volume of drainage. These findings align with studies from other surgical fields such as orthopedics and breast surgery [44,45,46], though similar studies in metabolic surgery remain lacking. Additionally, our study found that patients with elevated preoperative international normalized ratio (INR) values (>1) appeared to benefit more from TXA, showing a significant reduction in both hemoglobin concentration and mass in drainage samples. This finding suggests that TXA may be more effective and beneficial for patients with preoperative coagulation disorders, which is consistent with prior research [47,48,49].

The safety profile of TXA in our study was generally favorable, with no significant adverse events such as venous thromboembolism (VTE) observed at the 1-month follow-up. The literature reports a 30-day postoperative VTE rate from 0.14% to 1.9% in bariatric surgery patients [50,51]. Available research indicates that TXA does not significantly increase VTE incidence within 30 days post-surgery [52]. However, it is important to recognize that TXA is not without risks, including the potential for seizures, blurred vision, or skin rashes, and careful consideration should be given to its use in patients with predisposing factors for thromboembolic events, particularly in the context of obesity and bariatric surgery. In our study, no 30-day VTE events were recorded, although the study design and sample size preclude the conclusions regarding the safety profile of TXA administration [53]. The overall rate of VTE after abdominal surgeries is low, likely attributed to routine antithrombotic prophylaxis [54].

In conclusion, while TXA shows promise in reducing hidden blood loss after laparoscopic sleeve gastrectomy, its clinical significance in improving overall patient outcomes remains uncertain. The reduction in hemoglobin concentration in drainage samples is statistically significant but does not translate into meaningful changes in blood loss or clinical outcomes. Therefore, we do not recommend routine use of TXA in all bariatric surgery patients. However, it may offer benefits for selected high-risk patients, such as those with preoperative coagulation abnormalities or multiple metabolic syndrome components. Further research is needed to better define the optimal patient population and to explore the long-term benefits and potential risks of TXA in bariatric surgery.

### Limitations

This study has several limitations. The single-blind design may increase the risk of bias, while the single-center setting limited the sample sizes, necessitating further randomized clinical trials to validate these results. Additionally, no multi-center trials on TXA administration in metabolic surgery are available for comparison. This study may be underpowered to detect differences in AE rates or to fully establish the safety profile of TXA. Variability in TXA dosing across different studies further complicates direct comparison with existing research.

## 5. Conclusions

In conclusion, TXA administration mitigates the effects of hidden blood loss in bariatric surgery; however, its general clinical significance appears questionable, and its routine use within the ERABS protocol should not be recommended. Intraoperative use of TXA may be beneficial for a selected patient subgroup with multiple preoperative disorders and risk factors, although further research is required to comprehensively assess its risks and benefits. Both its efficacy and safety profile in a bariatric patient population should be further explored in a large multi-center randomized trial.

## Figures and Tables

**Table 1:** Baseline characteristics of the study groups (intention-to-treat and per-protocol analyses).

		Intention-to-Treat			Per-Protocol	
	CG (*n* = 123)	TG (*n* = 115)	*p*-Value for Group Comparison	CG (*n* = 102)	TG (*n* = 136)	*p*-Value for Group Comparison
Age (years)	41 [39.5–43.1]	41.4 [39.7–43.1]	0.926 ^1^	41.2 [39.4–43.1]	41.4 [39.7–43.2]	0.852 ^1^
Female gender	79 (64%)	77 (67%)	0.658 ^3^	63 (62%)	93 (68%)	0.288 ^3^
Height (m)	1.72 [1.71–1.74]	1.70 [1.58–1.90] *	0.753 ^2^	1.73 [1.71–1.74]	1.70 [1.58–1.90] *	0.112 ^2^
Preoperative weight (kg)	119 [90–172]	111 [86–175] *	0.097 ^2^	121 [91–172] *	111 [86–175] *	0.075 ^2^
Preoperative body mass index (kg/m^2^)	40 [33–53.1] *	38.9 [30.8–49.5] *	0.261 ^2^	39.9 [33.3–53.1] *	38.6 [31.4–49.8] *	0.167 ^2^
Maximal weight (kg)	135 [102–196] *	127 [94–190] *	0.046 ^2^	135 [104–196] *	127 [94–190] *	0.047 ^2^
Maximal body mass index (kg/m^2^)	45 [36.7–59.9] *	44.3 [35.9–56.8] *	0.089 ^2^	45.5 [37.8–59.9] *	44.1 [35.4–58.3] *	0.163 ^2^
Diabetes mellitus	21 (17%)	20 (17%)	0.948 ^3^	18 (18%)	23 (17%)	0.882 ^3^
Hypertension	54 (44%)	51 (44%)	0.945 ^3^	46 (45%)	59 (43%)	0.792 ^3^
Dyslipidemia	87 (71%)	77 (67%)	0.530 ^3^	76 (74.51%)	88 (65%)	0.106 ^3^
Previous abdominal surgeries	40 (33%)	43 (37.39%)	0.431 ^3^	31 (30%)	52 (38%)	0.209 ^3^
Preoperative lab tests						
Hemoglobin concentration (g/dL)	14 [13.9–14.4]	14 [13.8–14.3]	0.495 ^1^	14.5 [11.5–16.1] *	14 [13.8–14.2]	0.790 ^2^
Hematocrit (%)	43 [41.9–43.4]	42.3 [41.7–42.9]	0.481 ^1^	42.8 [42–43.6]	42.3 [41.7–42.9]	0.277 ^1^
Platelet count (10^3^/μL)	251 [174–394] *	263 [252.5–272.5]	0.746 ^2^	255 [174–388] *	263 [176–355] *	0.982 ^2^
White blood cell count (10^3^/μL)	8 [5–12] *	7.8 [7.4–8.1]	0.206 ^2^	7.8 [5–11.3] *	7.6 [4.9–11.4] *	0.396 ^2^
Hemoglobin mass (g)	875 [628–1261] *	843 [626–1280] *	0.206 ^2^	898 [628–1261] *	830 [626–1280] *	0.056 ^2^

For normally distributed continuous variables, data are presented as mean with a 95% confidence interval; otherwise, they are presented as median with a 5th–95th percentile range (*). Nominal variables are presented as N (percentage of the relevant group). Between-group differences were tested using ANOVA ^1^, Mann–Whitney U ^2^, and chi-square ^3^ tests. Preoperative hemoglobin mass was calculated according to Nadler’s formula [28].

**Table 2:** Postoperative outcomes (intention-to-treat and per-protocol analysis).

		Intention-to-Treat			Per-Protocol	
	CG (*n* = 123)	TG (*n* = 115)	*p*-Value for Group Comparison	CG (*n* = 102)	TG (*n* = 136)	*p*-Value for Group Comparison
Procedure time (min)	55 [40–90] *	54 [39–91] *	0.880 ^2^	55 [40–88] *	55 [40–95] *	0.408 ^2^
Postoperative lab tests from peripheral blood sample						
Hemoglobin concentration (g/dL)	13.5 [13.2–13.7]	13.5 [13.3–13.8]	0.751 ^1^	13.8 [10.9–15.4] *	13.5 [13.3–13.7]	0.407 ^2^
Hematocrit (%)	40.6 [39.9–41.4]	40.9 [40.3–41.5]	0.613 ^1^	40.7 [39.9–41.5]	40.8 [40.2–41.4]	0.873 ^1^
Platelet count (10^3^/μL)	255 [171–383] *	258 [247.4–268.6]	0.888 ^2^	257.5 [171–371] *	257 [162–346] *	0.913 ^2^
WBC count (10^3^/μL)	10.9 [10.4–11.4]	11.1 [10.6–11.5]	0.703 ^1^	10.9 [10.4–11.4]	11.1 [10.7–11.6]	0.437 ^1^
Difference in hemoglobin concentration (g/dL)	0.7 [0.5–0.9]	0.5 [0.4–0.7]	0.103 ^1^	0.7 [0.5–0.9]	0.5 [0.4–0.7]	0.089 ^1^
Difference in hematocrit (%)	2 [1.5–2.5]	1.4 [1–1.9]	0.072 ^1^	2.1 [1.6–2.6]	1.5 [1.1–1.9]	0.065 ^1^
Difference in platelet count (10^3^/μL)	1.5 [−4.1–7.1]	7 [−51–47] *	0.340 ^2^	3 [−2.9–8.9]	6 [−52–47] *	0.771 ^2^
Difference in WBC count (10^3^/μL)	−2.9 [−3.4–−2.5]	−3.3 [−3.7–−2.9]	0.245 ^1^	−3 [−3.4–−2.5]	−3.2 [−3.6–−2.8]	0.428 ^1^
Postoperative lab tests from drainage sample						
Drainage volume (mL)	40 [10–140] *	40 [5–101] *	0.187 ^2^	40 [5–160] *	40 [5–101] *	0.520 ^2^
Hemoglobin concentration (g/dL)	1.4 [0.1–7.1] *	0.7 [0.1–6.1] *	0.011 ^2^	1.7 [0.1–7.7] *	0.7 [0.1–6.1] *	0.001 ^2^
Hematocrit (%)	4 [0.1–22.9] *	1.8 [0.1–19.4] *	0.029 ^2^	5 [0.1–23.4] *	1.8 [0.1–19.4] *	0.003 ^2^
Hemoglobin mass (volume × concentration) (g)	0.4 [0–5.6] *	0.2 [0–3.4] *	0.005 ^2^	0.6 [0–9.6] *	0.2 [0–3.5] *	0.006 ^2^

For normally distributed continuous variables, data are presented as mean with a 95% confidence interval; otherwise, they are presented as median with a 5th–95th percentile range (*). Nominal variables are presented as N (percentage of the relevant group). Between-group differences were tested using ANOVA ^1^, Mann-–Whitney U ^2^, respectively.

## Data Availability

The data that support the findings of this study are not openly available due to sensitivity reasons and are available from the corresponding author upon reasonable request.

## Supplementary Materials

The following supporting information can be downloaded at: https://www.mdpi.com/article/10.3390/jcm14093010/s1, Table S1: List of complications; Material S1: Study protocol [1–10]; Material S2: Standard hospitalization protocol.
