# Supplementary material for: Effectiveness of Tranexamic Acid in Reducing Hidden Blood Loss During Laparoscopic Sleeve Gastrectomy: A Randomized Clinical Trial
Source: J Clin Med. 2025 Apr 26;14(9):3010. doi: 10.3390/jcm14093010 (PMC12072588; doi:10.3390/jcm14093010)
Supplement: Supplementary file 1 [file jcm-14-03010-s001.zip › Supplementary Material S1 - Trial protocol in accordance to SPIRIT guidelines.pdf]

Supplementary Material S1:

Clinical trial protocol (ver1.2) according to SPIRIT 2013 guidance.

Impact of Tranexamic Acid Administration on Postoperative Hemostasis in Laparoscopic Sleeve Gastrectomy: A Randomized Clinical Trial (TXA-SG).

The trial was registered in www.clinicaltrials.gov (NCT06038981).

This is a protocol version 1.2 (25.05.2022), some minor changes were incorporated: additional sub-analysis regarding influence of metabolic syndrome on hemoglobin concentration in drainage as well as sub-analysis for influence of preoperative INR on hemoglobin concentration in drainage.

This non-industry funded trial, therefore, no specific funding was assigned to the project.

All resources (equipment, drugs, services) will be provided by the local hospital participating in a trial.

K.B. and M.S. contributed to the conception of the study.

All authors contributed to the acquisition and interpretation of the data.

K.B. and M.S. contributed to the statistical analysis of the collected data.

K.B. prepared the tables and figures. K.B., M.S., M.P.-S., J.B., M.W. and P.N. drafted the manuscript.

All authors revised, critically reviewed and gave approval of the final version to be published.

Background:

Obesity and its metabolic outcomes has become an increasing problem for healthcare. Bariatric surgery is a well-established and safe method for the treatment of obesity and its complications (Alsumali et al., 2018; Noparatayaporn et al., 2021). However, bariatric surgery may be associated with adverse events such as nutritional deficiencies, anastomotic fistulas, gastrointestinal leaks, and haemorrhage (Kassir et al., 2016; McCarty & Kumar, 2022; Souche et al., 2018). Bleeding is one of the most common surgical complications in bariatric surgery. Reported rates of haemorrhage in literature vary from 0.5% to 4% (Kitahama et al., 2013). It is also associated with a significant increase in the complication rate (Wozniewska et al., 2021). Enhanced recovery after bariatric surgery (ERABS) implementation (Zhou et al., 2021) results in the quick mobilization of patients and a reduced length of hospital stay (LOS). Surgical hemostasis becomes even more important to achieve. Patients operated on according to the

ERABS protocol are, in general, discharged on the first postoperative day, unless there are reasons to keep them a day longer in hospital. Here, patient safety is one of the factors to consider. Postoperative bleeding is still one of the most common complications following sleeve gastrectomy (Mocanu et al., 2019) and is associated with higher mortality and morbidity (Straatman et al., 2022).

Amidst the rising prevalence of obesity and the proven effectiveness of bariatric surgery in managing obesity and its related metabolic conditions, optimizing postoperative outcomes and patient safety remains paramount. The concern of surgical bleeding, a common complication in bariatric surgery, highlights the urgent need for innovative solutions to mitigate this risk. Tranexamic acid (TXA), known for its antifibrinolytic properties, presents a promising intervention. There is a critical need for a well-structured clinical trial to investigate the influence of TXA on reducing hemorrhagic complications in patients undergoing bariatric surgery. This trial is vital to ascertain the efficacy of TXA in enhancing surgical hemostasis, thereby potentially lowering postoperative bleeding rates, improving patient recovery times, and reducing hospital stays. Such research could significantly contribute to advancing the safety and outcomes of bariatric surgery, offering a new horizon in the care of patients with obesity.

The primary study hypothesis is single dose of Tranexamic Acid will decrease hemoglobin concentration in abdominal drainage following sleeve gastrectomy and therefore, hidden blood loss will decrease.

The TXA-SG trial is designed as randomized, control, patient blinded single center trial with parallel groups, with primary endpoint of hemoglobin concentration in abdominal cavity drainage. The randomization will be performed as block randomization with allocation 1:1.

Study setting

This is a single center trial, conducted in academic hospital in a city of Gdansk, Poland.

Inclusion criteria

Patients eligible for the trial must comply with all the following at randomization:

- Age >= 18
- Qualification for sleeve gastrectomy as an initial procedure
- Obtained consent for participating in the trial.

Exclusion criteria:

- Qualification for a procedure other than SG
- Usage of anticoagulative agents in the perioperative period, including:

- Indirect thrombin inhibitors (Fondaparinux, UFH, LMWH in therapeutic doses)
- Direct inhibitors of factor Xa (NOAC)
- Direct thrombin inhibitors (Dabigatran)
- Vitamin K Antagonists (VKA: acenocumarol, warfarin)
- Platelet aggregation inhibitors (excluding ASA in doses of 75mg per day)
- P2Y12 receptor inhibitors
- Prior diagnosis of congenital or acquired blood coagulation disorders.
- Diagnosed allergic reactions to TXA in medical history.
- Chronic Kidney Disease in stage G3 or more advanced
- Chronic hemodialysis
- Haematuria in medical history
- Seizures in medical history

<u>Intervention for each group:</u>

All qualified for intervention patients will receive a single dose of 1g of i.v. TXA bolus at a loading dose of 1g over 10 minutes, within 10 minutes of the induction of anesthesia, preceding the anticipated skin incision.

Preceding the end of the procedure, a Redon drain will be placed alongside the staple line to monitor blood loss in the perioperative period. Drainage placement is performed only for study purposes.

Study protocol include single dose pharmacological intervention therefore there are no criteria for discontinuing of modifying allocated interventions.

The primary outcome parameter was the mean haemoglobin concentration in the drainage sample, measured through performing a CBC. The secondary outcomes were blood loss (defined as the difference between the preoperative and postoperative haemoglobin concentration in the peripheral blood sample), the drainage volume in ml, the haemoglobin mass in drainage (evaluated by multiplying the drainage volume and haemoglobin concentration) the procedure time (minutes), the extended postoperative hospital stay (days), major surgical complications due to bleeding (defined as a Clavien-Dindo score >=3), necessity for blood transfusion.

Enrolment starts on 4th of July, 2022; the power study calculation is scheduled after partial collection of data to evaluate study groups. The post operative follow-up visit is planned 30 days after intervention. The study will be conducted in the high-volume center (around 400 operations per year) therefore adequate enrolment will be ensured. A block randomization will

be used to allocate patients to different interventions based on the operating schedule of the center. Block size varies depending on the operating room availability. Patients will be assigned to the control and test groups alternately on a weekly basis. To further reduce possible bias, different physicians qualifying patients for operation who had no interaction with those physicians scheduling the operations. The collected data will be analyzed according to both the intention-to-treat and per protocol principles. Categorical data will be presented as numbers and percentages of the analyzed group. Continuous variables will be reported using mean and 95% confidence interval for normally distributed data and as median with 5th-95th percentile range otherwise. Statistical tests will be performed using SAS Studio ver. 3.81. Group comparison for categorical variables will be assessed using Chi-square test. ANOVA or Mann-Whitney U tests will be used for normally or non-normally distributed continuous variables respectively. The level of statistical significance will be set at $p<0.05$, 2-sided.

1. Alsumali, A., Eguale, T., Bairdain, S., & Samnaliev, M. (2018). Cost-Effectiveness Analysis of Bariatric Surgery for Morbid Obesity. *Obesity Surgery*, *28*(8). https://doi.org/10.1007/s11695-017-3100-0
2. Kassir, R., Debs, T., Blanc, P., Gugenheim, J., Ben Amor, I., Boutet, C., & Tiffet, O. (2016). Complications of bariatric surgery: Presentation and emergency management. *International Journal of Surgery (London, England)*, *27*, 77–81. https://doi.org/10.1016/J.IJSU.2016.01.067
3. Kitahama, S., Smith, M. D., Rosencrantz, D. R., & Patterson, E. J. (2013). Is bariatric surgery safe in patients who refuse blood transfusion? *Surgery for Obesity and Related Diseases : Official Journal of the American Society for Bariatric Surgery*, *9*(3), 390–394. https://doi.org/10.1016/J.SOARD.2012.02.008
4. McCarty, T. R., & Kumar, N. (2022). Revision Bariatric Procedures and Management of Complications from Bariatric Surgery. *Digestive Diseases and Sciences*, *67*(5), 1688–1701. https://doi.org/10.1007/S10620-022-07397-9
5. Mocanu, V., Dang, J., Ladak, F., Switzer, N., Birch, D. W., & Karmali, S. (2019). Predictors and outcomes of bleed after sleeve gastrectomy: an analysis of the MBSAQIP data registry. *Surgery for Obesity and Related Diseases*, *15*(10). https://doi.org/10.1016/j.soard.2019.07.017

6. Noparatayaporn, P., Thavorncharoensap, M., Chaikledkaew, U., Bagepally, B. S., & Thakkinstian, A. (2021). Incremental Net Monetary Benefit of Bariatric Surgery: Systematic Review and Meta-Analysis of Cost-Effectiveness Evidences. In *Obesity Surgery* (Vol. 31, Issue 7). https://doi.org/10.1007/s11695-021-05415-9
7. Souche, R., de Jong, A., Nomine-Criqui, C., Nedelcu, M., Brunaud, L., & Nocca, D. (2018). [Complications after bariatric surgery]. *Presse Medicale (Paris, France : 1983)*, *47*(5), 464–470. https://doi.org/10.1016/J.LPM.2018.03.024
8. Straatman, J., Verhaak, T., Demirkiran, A., Harlaar, N. J., Cense, H. A., Jonker, F. H. W., de Brauw, L. M., de Castro, S. M. M., Damen, S. L., Jonker, F., Dunkelgrün, M., Faneyte, I. F., Greve, J. W. M., Hazebroek, E. J., van ‘t Hof, G., Janssen, I. M. C., Jutte, E. H., Klaassen, R. A., Lagae, E. A. G. L., … Vening, W. (2022). Risk factors for postoperative bleeding in bariatric surgery. *Surgery for Obesity and Related Diseases*, *18*(8). https://doi.org/10.1016/j.soard.2022.05.010
9. Wozniewska, P., Diemieszczyk, I., & Hady, H. R. (2021). Complications associated with laparoscopic sleeve gastrectomy - A review. In *Przeglad Gastroenterologiczny* (Vol. 16, Issue 1). https://doi.org/10.5114/pg.2021.104733
10. Zhou, J., Du, R., Wang, L., Wang, F., Li, D., Tong, G., Wang, W., Ding, X., & Wang, D. (2021). The Application of Enhanced Recovery After Surgery (ERAS) for Patients Undergoing Bariatric Surgery: a Systematic Review and Meta-analysis. *Obesity Surgery*, *31*(3), 1321–1331. https://doi.org/10.1007/S11695-020-05209-5
