# Supplementary material for: Effectiveness of Tranexamic Acid in Reducing Hidden Blood Loss During Laparoscopic Sleeve Gastrectomy: A Randomized Clinical Trial
Source: J Clin Med. 2025 Apr 26;14(9):3010. doi: 10.3390/jcm14093010 (PMC12072588; doi:10.3390/jcm14093010)
Supplement: Supplementary file 1 [file jcm-14-03010-s001.zip › Supplementary Material S2 - Standard operative and hospitalization protocol.pdf]

Supplementary Material 2:

Standard perioperative protocol for bariatric surgery.

Patients are admitted to the surgical ward one day before the operation (long-track protocol) in the case of the distance to hospital being more than 50 km or patients presenting movement disabilities or of eldery age, otherwise the admission takes place on the day of surgery, in the morning (fast-track protocol - 7:00). During admittance, a physical examination and anamnesis are performed together with rework of the lab tests. The standard premedication is used which includes oral admission of 1 g of Paracetamol, 20 mg of Omeprazolum and 10 mg of Metoclopramidum. Additionally, for antibiotic prophylaxis, a weight dependent dose (1-3 g) of Cefazolinum is administered.

The surgery is conducted under general anesthesia according to the standardized protocol, which includes the medication set: sufentanil 10 mcg i.v.; propofol 1.5-2 mg/kg IBW i.v.; rocuronium 0.6 mg/kg IBW or, for consideration, succinylcholine in cases of anticipated difficulties in tracheal intubation. During the initial phase of the procedure, the patient receives dexamethasone 8 mg i.v. and metamizole 2.5 g i.v. in an infusion lasting about 30 minutes. At the end of the procedure, oxycodone 10 mg i.v. is administered. During an uncomplicated surgical procedure, balanced, restrictive fluid therapy is recommended. During anesthesia, the use of coanalgesics such as IV ketamine, IV magnesium, IV paracetamol or IV metamizole is allowed. Reversal of the action of neuromuscular blocking agents is performed using atropine in a dose of 0.5 mg i.v. and neostigmine 1.0-3.0 mg i.v. or sugammadex 2 mg/kg IBW.

We used the four-trocar technique to access the abdominal cavity, Hasson technique was used to establish the pneumoperitoneum (14 mmHg). The LigaSure™ device is used for liberating the greater curvature of a stomach. A calibrating tube is used for creating a gastric sleeve (34-36 Fr). Medtronic Endo GIA™ TRI-staple™ technology is used in the following order: X-Tra thick (black) cartridge, followed by two Medium/Thick (purple) cartridges followed by Vascular/Medium (gold) (the number of gold staples depends on the length of the gastric sleeve). Routine endoscopic leak test is not performed.

In postoperative pain management the following painkillers are given intravenously: 4x1 g of Paracetamol and 4x1g of Metamizol (in 3-hour intervals each). In case of an increased need for painkillers (NRS >-6), patients temporarily receive 100 mg of intravenous Tramadol (up to a maximum dose of 400 mg per day) or 10 mg of intravenous Oxycodone.

To facilitate postoperative nausea, vomiting and flatulence, 40 mg of Drotaverin, 10 mg of Metoclopramidum, 8 mg of intravenous Ondansetron or 8 mg of intravenous Dexomethasone

could be prescribed. At 5pm on the first day after surgery, standard laboratory tests are taken from peripheral blood samples, which include: glucose, sodium, potassium, CBC, and C-reactive protein. According to our discharge protocol, every patient needs to fulfill all discharge criteria: low/moderate pain, ability to drink and swallow medicaments, general good condition, no tachycardia. Before being discharged, bariatric education, including dietary recommendations in the first days post-surgery, is conducted. Patients are also informed about wound care management. They leave the ward with a referral for an outpatient clinic with the appointed day and time of the first control visit, which takes place after 7-14 days following surgery. Then, the next appointment is scheduled for approximately one month after surgery. Patients are obligated to do a lab test before the appointment.
