# Supplementary material for: Effectiveness of Tranexamic Acid in Reducing Hidden Blood Loss During Laparoscopic Sleeve Gastrectomy: A Randomized Clinical Trial
Source: J Clin Med. 2025 Apr 26;14(9):3010. doi: 10.3390/jcm14093010 (PMC12072588; doi:10.3390/jcm14093010)
Supplement: Supplementary file 1 [file jcm-14-03010-s001.zip › Supplementary Table S1 - List of complications.pdf]

Supplementary Table 1: List of complications.

Group:	Clavien-Dindo:	Complication - description:
CG	3A	Trocar site hematoma
	3A	Staple line leakage, endoscopic treatment with MEGA stent implantation
	3B	Endoscopic widening of esophagogastric junction
	3B	Reoperation due to bleeding, staple line hemostasis
	3B	Reoperation due to bleeding, staple line hemostasis
	3B	Staple line leakage, intraabdominal abscess - reoperation, abdominal cavity lavage and drainage, treatment with MEGA stent implantation. After 6 weeks observation and stent removal - diagnosis of esophagopleural fistula.
TG	3B	Staple line leakage, endoscopic treatment with MEGA stent implantation
	3B	Staple line leakage, endoscopic treatment with MEGA stent implantation
	3B	Reoperation due to bleeding - staple line hemostasis, abscess in the central trocar site

CG – control group; TG – test group.
